# A Feasibility Study of Remote Non-Contact Vital Signs (NCVS) Monitoring in a Clinic Using a Novel Sensor Realized by Software-Defined Radio (SDR)

**DOI:** 10.3390/bios13020191

**Published:** 2023-01-27

**Authors:** Yang Liu, Clint Sweeney, Jill C. Mayeda, Jerry Lopez, Paul E. Lie, Tam Q. Nguyen, Donald Y. C. Lie

**Affiliations:** 1 Department of Electrical and Computer Engineering, Texas Tech University, Lubbock, TX 79430, USA; 2 Texas Tech University Health Sciences Center (TTUHSC), Texas Tech University, Lubbock, TX 79430, USA

**Keywords:** biosensor, COVID-19, non-contact vital signs (NCVS), respiration rate (RR), software-defined radio (SDR), telemedicine, wireless acute care, wireless assisted living

## Abstract

The COVID-19 outbreak has caused panic around the world as it is highly infectious and has caused about 5 million deaths globally. A robust wireless non-contact vital signs (NCVS) sensor system that can continuously monitor the respiration rate (RR) and heart rate (HR) of patients clinically and remotely with high accuracy can be very attractive to healthcare workers (HCWs), as such a system can not only avoid HCWs’ close contact with people with COVID-19 to reduce the infection rate, but also be used on patients quarantined at home for telemedicine and wireless acute-care. Therefore, we developed a custom Doppler-based NCVS radar sensor system operating at 2.4 GHz using a software-defined radio (SDR) technology, and the novel biosensor system has achieved impressive real-time RR/HR monitoring accuracies within approximately 0.5/3 breath/beat per minute (BPM) on student volunteers tested in our engineering labs. To further test the sensor system’s feasibility for clinical use, we applied and obtained an Internal Review Board (IRB) approval from Texas Tech University Health Sciences Center (TTUHSC) and have used this NCVS monitoring system in a doctor’s clinic at TTUHSC; following testing on 20 actual patients for a small-scale clinical trial, we have found that the system was still able to achieve good NCVS monitoring accuracies within ~0.5/10 BPM across 20 patients of various weight, height and age. These results suggest our custom-designed NCVS monitoring system may be feasible for future clinical use to help combatting COVID-19 and other infectious diseases.

## 1. Introduction

More than two years have passed since the first COVID-19 case was reported at Wuhan city, China. However, even with the help of vaccines, this global pandemic is still not under good control. New variants of the virus, such as the delta variant, delta plus variant, lambda variant, omicron variant, etc., continue to pop up. To help protect healthcare workers (HCWs), remote monitoring of vital signs using various techniques such as visible light sensing [1], millimeter wave radar [2], UWB (ultra-wideband) radar sensor [3], phased-array and fixed beam CW (continuous-wave) radar sensors using custom hardware [4,5], and software-defined radio (SDR)-based radar sensor systems have all been reported [6,7,8,9,10,11]. Differently from traditional contact-based vital signs monitoring systems currently widely used in clinically practices, these remote or non-contact vital signs (NCVS) monitoring systems are targeted to monitor patients’ vital signs remotely and continuously; therefore, if an NCVS sensor system can be proven to be robust enough for clinically use, it would offer some key advantages as follows: (1) the HCWs can avoid direct contact with people infected with COVID-19 during vital signs setup/adjustment/reading to reduce their infection risks; (2) the NCVS monitoring system can be used on people suspected to be or confirmed infected with COVID-19 to safely quarantine them at home or in a hotel for telemedicine practice and for wireless acute-care. In this work, we focus our discussions on an SDR-enabled NCVS sensor system, where the continuously monitored vital signs data can be easily digitalized, analyzed and wirelessly transmitted to update and alarm HCWs on patients’ status. It may also potentially help to significantly increase patients’ vital signs monitoring outside of the Intensive Care Unit (ICU) setting.

A patient’s vital signs are among the most important information checked and monitored by the HCWs; they are a group of four to six key measured bio-signals that indicate the status of a person’s basic life-sustaining functions. The four major vitals usually checked first by the HCWs are: body temperature, pulse rate (e.g., heart rate, HR), respiration rate (e.g., breathing rate, BR or RR), and blood pressure (e.g., BP; note that strictly speaking, BP is not considered a vital sign, but is often measured along with the vital signs). An abnormal body temperature could mean systemic inflammation or illness. HR represents how fast one’s heart beats and is often shown in the unit of beats per minute (BPM). RR shows how many breaths one takes per minute. A resting RR under 12 or over 25 breaths per minute is usually considered abnormal for adults [12]. Among the conditions that can cause abnormal RR are asthma, apnea, anxiety, pneumonia, COVID-19, congestive heart failure, lung diseases, narcotics, sedation, etc. Clinically, high RR on admission is highly correlated with in-hospital mortality rate for COVID-19 patients [13], and abnormal RR also affects recovery in the post-anesthesia care unit (PACU) [14].

Among the various NCVS technologies mentioned above, visible light sensing [1] technology is based on analyzing the power change in the received signal due to the periodic chest movement generated with respiration and heartbeats, whereas the frequency-modulated continuous wave (FMCW) radar [2] method processes the IF (intermediate frequency) signal, which contains information about the chest movement. The UWB radar [3], on the other hand, directly measures the periodic distance change in chest movement to estimate the RR and HR. As for the SDR-based radar sensor systems, several studies have focused on the phase changing of the received signals [6,7,8,9], while one work also targeted the distance variation [10], and the other extracted the RR and HR by analyzing the change in both phase and magnitude of the received signal [11].

Differently from those reported NCVS sensing systems, our group designed a novel SDR-based CW radar system which can calculate both the RR and HR by analyzing the received and downconverted RF signals phase modulated by the periodic respiration and heart movements. Our group has reported an SDR-based prototype radar sensor system for NCVS monitoring by using a commercially available USRP-2901 (from National Instruments, NI) SDR along with codes written in LabView programming. Using a robust universal SDR box hardware with a mature software development environment (i.e., LabView) provides some significant advantages to speed up prototype development and potential future clinical deployment, as well as offering good system reliability and easy functionalities updates. Our initial NCVS system utilized a homodyne receiver architecture [7], and then further improvement to the system’s robustness is achieved by using a low-IF (intermediate frequency) receiver architecture with excellent RR and HR monitoring accuracy and optimized gain settings [8]. The Low-IF NCVS system is currently being used in a clinical trial at TTUHSC to further test the system’s accuracy and limitations, as the results will be reported in this paper. The details of NCVS sensor system operation principles, measured performance on student volunteers at our engineering lab testing, and the latest testing data on real patients during the clinical trial will be discussed next. However, there are still many challenges to overcome in order to make this SDR-based NCVS system useful for practical use. Therefore, this current small-scale clinical trial measurement setup (for a monitored patient to be sitting still in a chair in the clinic) is only our 1st step to demonstrate the system’s basic capability on actual patients, as they come in with different body types and ages, etc. Therefore, this current trial is not meant for the demonstration of continuous remote non-contact vital signs monitoring yet; for that, a wearable sensor would be preferred, but note that wearable sensors alone are not desirable for patients with COVID-19 or other highly infectious diseases, as the setup and adjustment of those contact-based wearable sensors require close patient contact, therefore exposing the clinicians to potential serious infections and unnecessary danger. Accurate remote monitoring of patients’ RR is the key goal for our NCVS system presented in this work, as that can be very effective in accessing patients’ real-time respiration status to help combatting patients with COVID-19 or other serious respiration and/or infectious diseases. NCVS monitoring may thus be very useful to complement the traditional contact-based wearable sensors for vital signs monitoring in certain cases.

## 2. NCVS Sensor System Operation Principles

**Figure 1:**
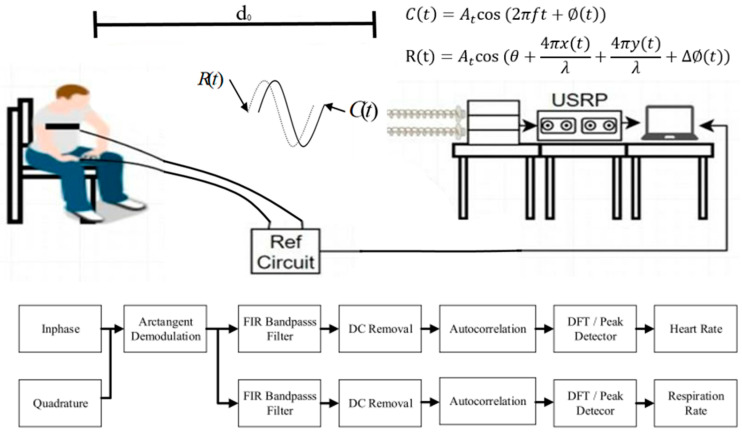
Basic experimental setup of our Doppler-based NCVS detection system using the NI SDR, and the block diagrams showing how to extract the vital signs information [5].

The system built with the SDR kits is a continuous wave (CW) Doppler based NCVS sensing system. Figure 1 illustrates the basic ideas of the experimental set up of the system and its operational principles. The transmitted CW signal is *C(t)*, and the reflected and received CW signal is *R(t)* in Figure 1, where *d*_0_ is the distance between the antennas and the subject. Since the subject’s heart beats and chest wall movements are periodic, these movements produce no net velocity and would not cause a Doppler frequency shift in the signal, but instead, they produce a phase modulation. Instead of deriving this relationship rigorously, one recalls the Doppler equation on frequency shift due to the target velocity $f_{d}\left(t\right)=\frac{2f}{c}v\left(t\right)=\frac{2v\left(t\right)}{\lambda }$, where *v(t)* is the subject’s velocity. If the target only has periodic movement, it has no net velocity and hence will produce no Doppler frequency shift. However, one can integrate the Doppler equation above in the time domain to obtain $\phi \left(t\right)=\frac{2f}{c}\left(2\pi x\left(t\right)\right)=\frac{4\pi x\left(t\right)}{\lambda }$, where *x*(*t*) is the displacement of the chest. The CW signal *R(t)* reflected back from the chest of the monitored subject due to the periodic chest movement *x(t)* is thus phase modulated and contains both the breathing rate information and the heart rate information, with the breathing rate being the lower-frequency component and the heart rate being the higher-frequency component (filter range from 0.05 to 2 Hz used in our system). The reflected signal *R(t)* can be expressed with the equation $R\left(t\right)=cos\left(\theta +\frac{4\pi x\left(t\right)}{\lambda }+\frac{4\pi y\left(t\right)}{\lambda }+\Delta \emptyset \left(t\right)\right)$, where *x(t)* is the breathing rate signal, *y(t)* is the smaller heartbeat signal, θ is the constant phase shift dependent on the distance from the target *d*_0_, and Δ∅(t) is dependent on the phase noise of the VCO (voltage controlled oscillator) or PLL (phase-locked loop) used in the NCVS system [5]. However, just like a typical Doppler radar, if one only uses 1 channel, it will have null points at every quarter wavelength, where it would be difficult to obtain reliable vital signs readings. We are using a CW signal at 2.4 GHz, so the wavelength in the free space is approximately 12.5 cm, which gives us a null point at every 3.125 cm. To get around this issue, we use the quadrature I/Q channels of the NI2901 SDR in combination with arctangent demodulation. Sending the received signal through the quadrature architecture and processing while ignoring the DC offset and channel mismatches, we obtain $I=cos\left(\theta +\frac{4\pi x\left(t\right)}{\lambda }+\frac{4\pi y\left(t\right)}{\lambda }+\Delta \emptyset \left(t\right)\right)$ and $Q=sin\left(\theta +\frac{4\pi x\left(t\right)}{\lambda }+\frac{4\pi y\left(t\right)}{\lambda }+\Delta \emptyset \left(t\right)\right)$. These functions are then put through the function $\emptyset =tan^{-1}\left(\frac{Q}{I}\right)$ in software, which results in $\emptyset =\left(\frac{sin\left(\theta +\frac{4\pi x\left(t\right)}{\lambda }+\frac{4\pi y\left(t\right)}{\lambda }+\Delta \emptyset \left(t\right)\right)}{cos\left(\theta +\frac{4\pi x\left(t\right)}{\lambda }+\frac{4\pi y\left(t\right)}{\lambda }+\Delta \emptyset \left(t\right)\right)}\right)=\theta +\frac{4\pi x\left(t\right)}{\lambda }+\frac{4\pi y\left(t\right)}{\lambda }+\Delta \emptyset \left(t\right)\;$as shown in Figure 1. As the arctan data is used to extract the measurement HR/RR, we apply a real-time DC removal process to it by subtracting the mean value of the stored real-time arctan dataset. The refresh rate for the system is 25 Hz, and the sample rate is 200 Hz, Thus, the system will be updated with the measurement HR/RR every 0.04 s, and every 0.04 s, there will be 8 data points added into the database, which also means the mean value of the arctan dataset will be updated every 0.04 s. Therefore, from this time-domain signal of the periodic displacement of the heart and the chest wall, one can extract the heart rate and respiration rate at the same time by applying filtering, DC removal, autocorrelation, DFT (Discrete Fourier Transform) and peak finding of the heartbeat and respiration signals in the frequency domain [5].

## 3. Testing at Our Engineering Lab with Young Student Volunteers

**Figure 2:**
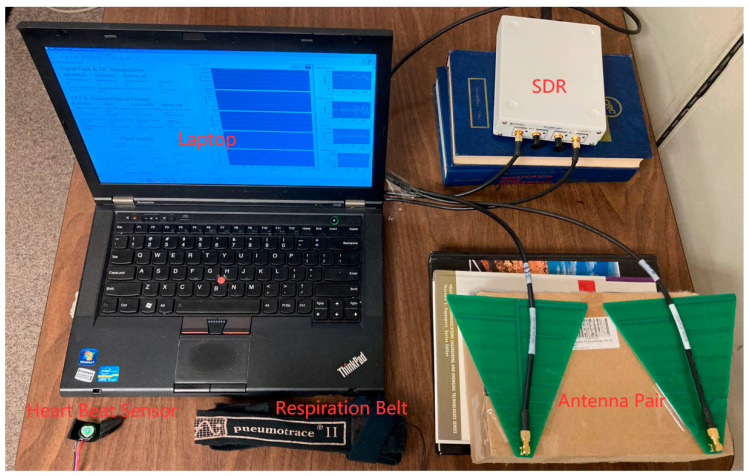
The NCVS hardware components and antennas placement.

Figure 2 shows the hardware portion of our SDR-based NCVS system. It includes a laptop, an SDR unit, and a pair of Log-Periodic Dipole Antennas (LPDA). To compare with the vital signs measured by the traditional contact-based sensors as reference, only Figure 2 also includes a respiration belt to wrap around the subject’s chest to produce the reference RR. The respiration belt is a solid-state transducer which measures respiratory changes in the chest or abdominal circumference. It is a piezo-electric device, which responds linearly to changes in elongation, generating a positive voltage as the length increases, and the output with respiration can be anywhere between 20 and 400 mV [15]. By analyzing this periodic voltage signal changing due to the chest movement using FFT (Fast Fourier Transform) in LabView by finding the peak frequency converting to BPM, the reference RR is obtained. Additionally, a heart rate sensor is placed on the subject’s fingertip to produce the reference HR signals. This is an optical sensor, which uses light to measure tissue changes at the sensor’s location caused by blood circulating throughout the body. As one’s heart beats, this volume changes and the higher blood volume causes less light to return to the optical sensor, whereas the lower volume increases the amount of returning light [16]. The laptop should have enough random-access memory (RAM) to run the LabView code that controls the SDR and performs the data analysis. The SDR being used here, the NI USR-2901 [17,18], which generates the 2.4 GHz transmit signal, filters and amplifies the received signal and downconverts it to IF, and then digitizes the signal according to the programmed parameter settings on the laptop. A pair of printed circuit board (PCB) LPDA antennas with a gain of 6 dBi at 2.4 GHz, a bandwidth of 0.9–6 GHz, and a size of 12 cm × 15 cm are used in the system [19]. They are placed at such an angle that the receive (RX) antenna can receive the reflected transmit (TX) signal from the monitored subject, and that there is sufficient distance separating the TX/RX antennas to reduce their interactions. 

**Figure 3:**
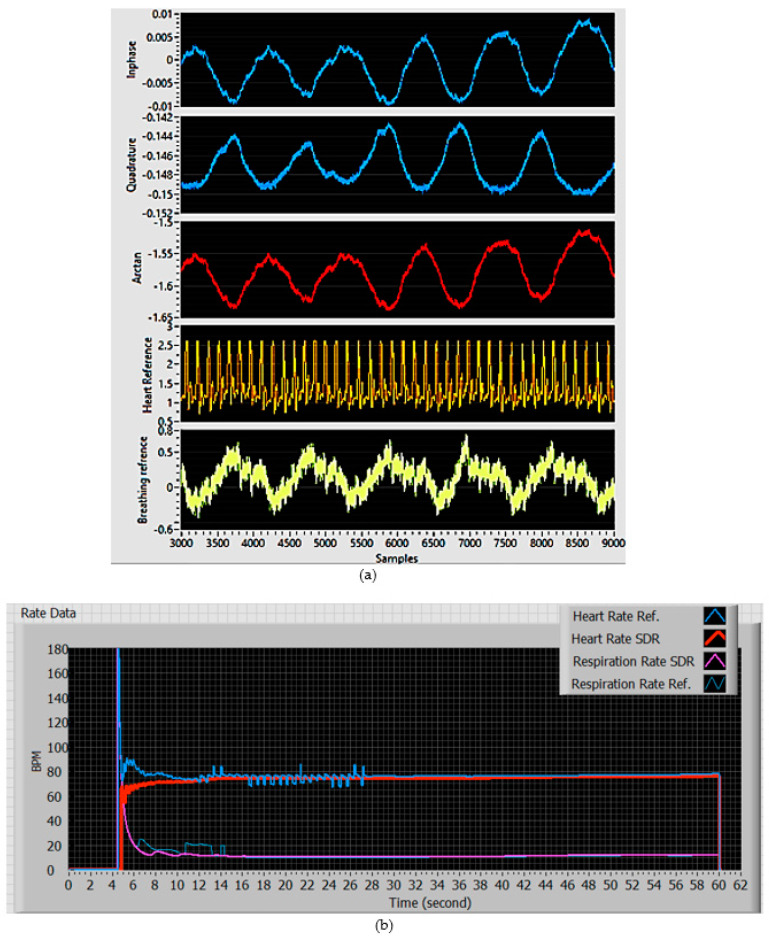
The core section of the GUI of the NCVS system: (**a**) I/Q/Arctan waveforms (top 3 channels) and the reference HR/RR signal waveforms (bottom 2 channels) all in the time domain; (**b**) the real−time NCVS system−measured HR/RR and the measured HR/RR from the reference sensors all plotted vs. time.

The fundamental operational principle of our Doppler-based NCVS system has been described in [4,5,6,7] and above. Basically, our system transmits a 2.4 GHz continuous wave (CW) RF signal and then receives the reflected wave off the subject’s chest wall. The periodic chest movement (caused by respiration and heartbeats) returns a phase-modulated signal that can be extracted into vital sign signals, by applying FFT, filtering, peaks finding, etc., as shown in Figure 1. Figure 3a displays the core section of our NCVS sensor system’s graphic user interface (GUI), which shows the Inphase (I), Quadrature (Q), and Arctan channels’ waveforms measured by the NCVS sensor system (i.e., the top 3 channels), followed by the measured reference HR and RR waveforms from the contact-based sensors (i.e., the bottom 2 channels). Figure 3b shows the real-time recording of NCVS system HR/RR measured results vs. the reference HR/RR values. Additionally, to optimize and adjust the NCVS sensor system’s performance, the TX/RX gain setting, system operating frequency, IF frequency, I/Q rate, testing time duration, etc. can all be changed in this GUI.

**Figure 4:**
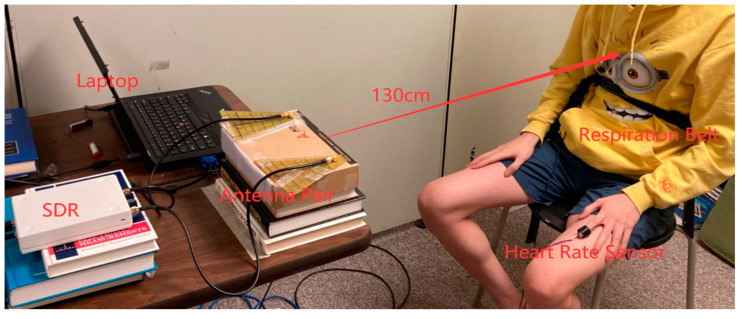
Example lab testing setup with a student volunteer sitting on a chair placed 130 cm away.

To start testing our NCVS monitoring system in our lab on young student volunteers, one can put the contact-based reference sensors on, sitting on a chair placed 130 cm away from the end of the antennas, and then run the LabView program as shown in Figure 4. The system will stop monitoring and recording automatically after the test has run its course. During testing, the subject should sit still without significant body movement, since this Doppler-based NCVS sensor system operates by detecting the periodic change in phase according to chest displacement caused by heartbeat and respiration movements [9]; any other kind of body movement that occurs during a test may decrease the system’s accuracy.

**Figure 5:**
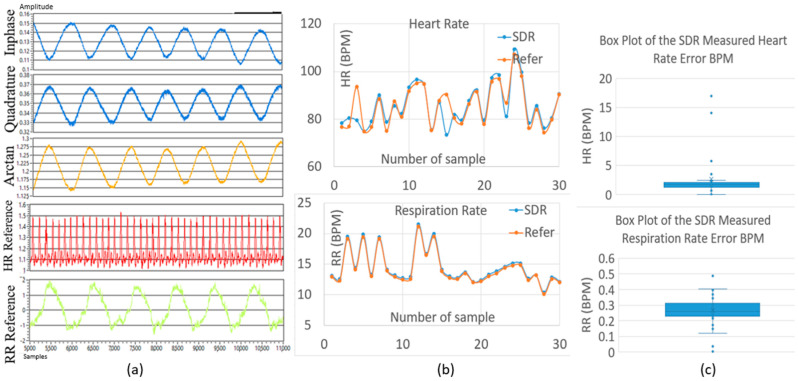
Example of output waveforms and test data and errors from the NCVS monitoring of 30 test cases gathered from 7 student volunteers with optimized NCVS system settings at our engineering lab: (**a**) measured I/Q/Arctan waveforms with reference HR/RR signals; (**b**) NCVS−measured HR/RR data vs. reference sensor data; (**c**) HR/RR errors in boxplots vs. reference signals.

The system parameters need to be set carefully to obtain clear channel waveforms and accurate output data. For example, the TX/RX “gain pair” affects the power of the transmitted/received signal, and a mismatched gain pair might result in the received signal levels being too high or too low, which may result in noisy channel waveforms and inaccurate output data. The low-IF architecture is applied here to reduce the 1/f noise, since the frequency of the HR/RR signals are usually rather close to DC and lower than 2.5/0.5 Hz, respectively (e.g., the HR/RR is lower than 150/30 BPM). Thus, the system may suffer from huge 1/f noise if the received signal was directly downconverted to baseband [20]. Since this NCVS sensor system can produce rather accurate RR at 1 m and 1.5 m in our engineering lab, with average error rates of 0.64 and 0.26 BPM, respectively [6], in this work, we fix the test range at 1.3 m to optimize the system parameters setting. After performing experiments to optimize the system performance by adjusting various parameters in our engineering lab, the TX/RX gain and IF frequency were found to be optimal when set to 34/45 and 1 kHz at the system operating frequency of 2.4 GHz. Figure 5 shows an example of the channel waveforms and 30 test results obtained from 7 student volunteers. There are two cases that show large heart rate errors of 15 BPM and 17 BPM, respectively. As the signal from the heart beats is rather weak compared to the signal from the larger respiration movements, the HR measurement data is considerably more susceptible to interferences and noises from: (1) the harmonics of the respiration rates [21]; (2) the random movements of the subject during monitoring [22]; and (3) background movements (people moving nearby, floor vibrations, air flows, etc.) [23]. In the practical monitoring environment, therefore, it would be very challenging to always keep the remote monitored HR data accurate. However, it would be much easier to obtain accurate RR data, as our work presented here has demonstrated, both in the engineering lab and also on real patients. Accurate remote monitoring of patients’ RR is the key goal for our NCVS system presented in this work, as that can be very effective in accessing patients’ real-time respiration status to help combatCOVID-19 or other serious respiration diseases remotely. In general, this data indicates that the NCVS system can continuously monitor the real-time RR/HR 1.3 m away, confining errors consistently within 0.5/3 BPM, respectively, compared with the reference RR/HR signals. It also produces clear channel waveforms in the lab when the optimized settings were applied.

**Figure 6:**
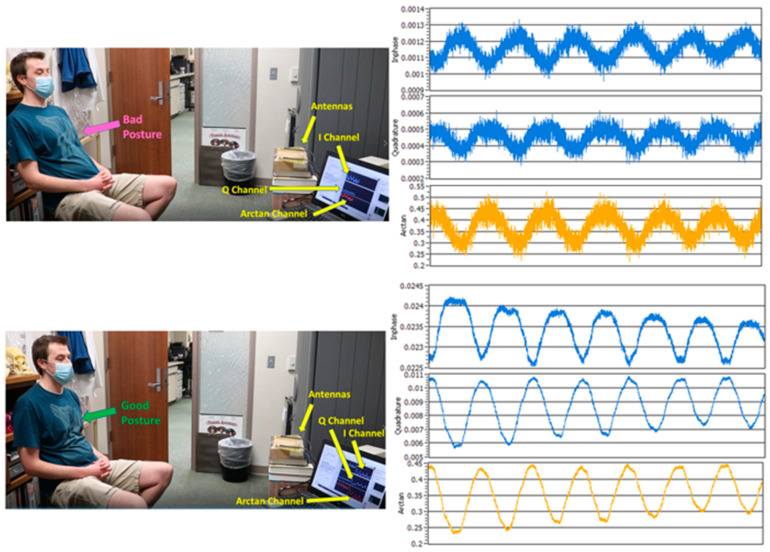
Plots showing a subject’s posture affects the quality of output waveforms for the NCVS sensor system and the vital signs monitoring accuracies.

**Figure 7:**
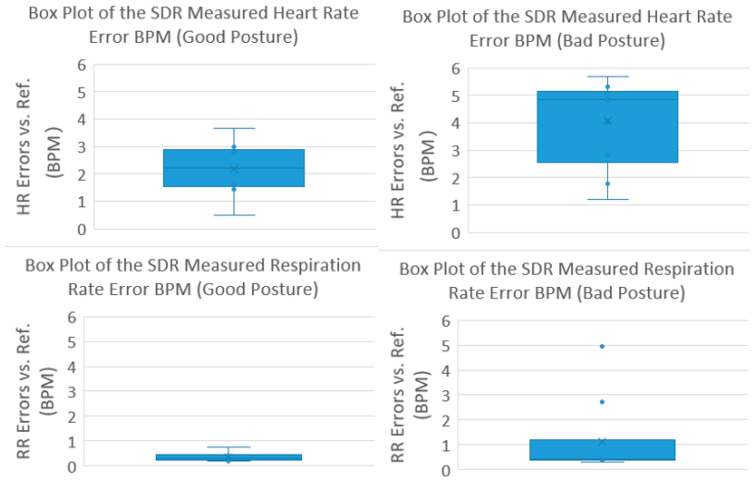
Analysis of output data of 10 cases of student volunteers with “Bad posture” vs. “Good posture”.

Besides the system parameter settings, a patient’s sitting posture should also be considered when attempting to obtain clear waveforms and accurate NCVS output data. Figure 6 shows the examples of “Good” and “Bad” postures, and the corresponding channel waveforms. If a subject changes his/her posture from “Bad Posture” to “Good Posture”, as shown in Figure 6, it will help the NCVS sensor system to produce clearer channel waveforms with better signal-to-noise ratio (SNR). The main reason is that when a volunteer is sitting in a laid-back “Bad Posture” position on the chair, the reflected signal may not directly go to the receiver antenna, resulting in decreased power of the received signal and thus reducing the SNR of the waveforms and degrading the vital signs data quality to be extracted. When the volunteer sits up straight in a “Good Posture”, shown in Figure 6, the power of the received signal becomes stronger, making the Arctan signal clearer with better SNR. Thus, the I/Q/Arctan channel waveforms from a subject with a bad posture would look noisier compared to those from a subject with a good posture. As the I/Q channel waveforms are voltage waveforms, we can roughly calculate the SNR difference between the “Bad” and “Good” postures. In Figure 6, for the “Bad” posture, the voltage swings on the I/Q channels are about 0.0003 and 0.00015 V, respectively. However, for the “Good” posture, they are about 0.0015/0.004, respectively. Since the combined magnitude for the I/Q channels can be calculated as $\frac{\sqrt{I^{2}+Q^{2}}}{2}$, and the voltage signal level for the “Good” posture is about 5–10 times higher than that of the “Bad” posture, this will translate to a better SNR of 14 to 20 dB, which is considerably better. Testing results for 10 cases are shown in Figure 7 with student volunteers in the TTUHSC clinic, where the clinical trial is taking place. For subjects with “Bad Posture”, the error of HR/RR is about 4/1 BPM, respectively, whereas for subjects with “Good Posture”, the error is reduced to 2.5/0.5 BPM, respectively. The measurement HR/RR data also has wider fluctuations for cases with “Bad Posture” compared to cases of “Good posture”. Both phenomena indicate that a subject with “Good Posture” indeed improved NCVS monitoring accuracy. As people have different body types and different height, weight, preferred sitting postures, etc., it may be difficult to set a universal criteria on “Good Posture” for every patient. However, what we did in practice to help the NCVS system obtain better accuracy was to look at the real-time I/Q/Arctan waveforms during the initial measurement; if the waveforms look clear (not fussy with noisy waveforms or distortion) and are of sufficient signal strength (say > 3 mV), the posture is an acceptable “Good Posture”, especially good enough for RR monitoring in practice.

## 4. Test Results Comparison with the Literature

As reported by [1,2,3,7,9,10,11] and briefly discussed in the Introduction, several proposed NCVS sensing methods have been reported in the literature. Note, for example, that VPG (videoplethysmographic) systems may apply multichannel photoplethysmographic imaging to remotely measure the HR and RR, and some can even measure blood pressure. However, since it may be more invasive to patients’ privacy than a radar-based NCVS system, we did not include this technique in our clinical trial study here [24]. The accuracy of visible light NCVS sensing is more than 94% for both HR and RR measurements [1]. An FMCW radar study reported an accuracy better than 90% for both HR and RR [2], and a study using a UWB radar sensor has an RR accuracy of more than 95%, while the HR accuracy is around 90% [3]. The accuracy of SDR-based CW radar systems with different HR and RR extraction methods varies between 80% and 95% [9,10,11]. 

In addition, an SRD-based system has greater capability to work in different scenarios. Firstly, the antenna is replaceable, which means that other than the LPDA we used in this work, a helical antenna can also be used for long range monitor as it has higher directivity. Next, the system parameters such as TX/RX gain, I/Q rate and IF are easily tunable; by adjusting these system parameters, the system performance can be optimized for different range monitoring, as [6] presents, wherein the HR monitoring is performed at 1 m and 1.5 m, and the average error rate are as low as 0.64/0.26 BPM, respectively. Moreover, the SDR-based system can provide a competitive solution for NCVS monitoring applications, especially for remote RR monitoring, as it has been shown to be accurate and easy to setup and can provide the monitoring range greater than millimeter-wave solutions and at a frequency that can penetrate thick blankets and clothes. The SDR box is not a custom-made hardware as many other published CW and FMCW radar NCVS systems are, which made these SDR boxes attractive to us for clinical trials. Note that our group has designed five generations of custom radar hardware, but once students graduate, it becomes difficult to re-make those hardware for clinical trials [25,26].

Table 1 shows our SDR-based NCVS system has achieved an HR/RR accuracy performance that ranks among the best reported in the literature on young healthy volunteers. The obtained good NCVS monitoring accuracy results are shown in Table 1.

Moreover, IWR6483AoP (antenna on package) [27] is an on-chip radar sensor system developed by Texas Instruments (TI) which has the capability to monitor the HR and RR remotely. Compared to the SDR we are using [7,8], IWR6483 has higher operating frequency 60–64 GHz and higher max I/Q rate at 25 MS/s which helps it to handle higher IF. This product also implements a 4 × 3 phased array system [28].

The basic theory of the remote HR/RR monitoring is using FMCW radar to create an IF signal [29], where the IF signal will have periodic phase changes due to subject’s chest movements. Thus, similarly to what we have developed here, by applying FFT and peak finding to this IF signal, the HR and RR can be extracted. The major limitation of this powerful millimeter-wave radar system is the monitoring distance and how well it can penetrate thick blankets and clothes, and with a greater monitoring distance, a higher IF will be generated, then a higher I/Q rate will be required. It is reported that the suggested range is between 0.1 and 0.7 m; however, the HR/RR accuracy values are not provided in that ref. [29].

## 5. Clinical Trial at TTUHSC

**Figure 8:**
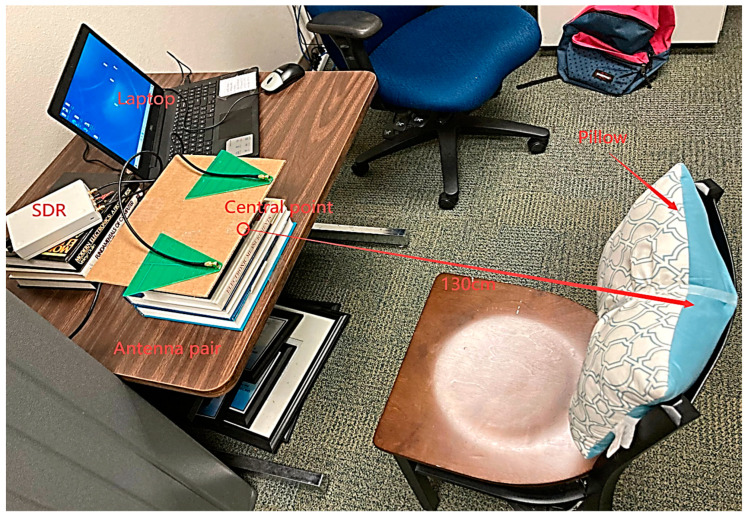
An example of the clinical trial setup of our NCVS system in a room of the TTUHSC Physicians Clinic.

The lab testing results showed the robustness of this NCVS system during its research phase on student volunteers. To further test it in the real word, a clinical trial on actual patients is required and planned. We have thus applied for an IRB (Internal Review Board) to start a clinical trial at the Physicians’ Clinics of Texas Tech University Health Sciences Center (TTUHSC), and it was approved in the 1st half of 2021. Unfortunately, the clinical trial period had to be reduced by more than what we originally planned, due to a sudden increase in COVID-19 cases and additional training required, but we managed to obtain data for 20 patients, and the results will be presented here. As it is a non-contact monitoring system, the contact-based RR/HR reference sensors (the respiration belt and the finger-tip heart rate sensor) were removed during the clinical trial. A graduate student was in the room with the patient to remind the patient to sit straight and also to operate the laptop to start the measurement and to save the data. Figure 8 shows an example of the clinical trial setup in a TTUHSC’s clinic room. The chair is placed 1.3 m away (the same as in the lab testing); however, after considering the posture issues, a pillow was attached on the back of the chair that to help the patients feel comfortable, so that they could sit still and sit up straight during the test.

**Figure 9:**
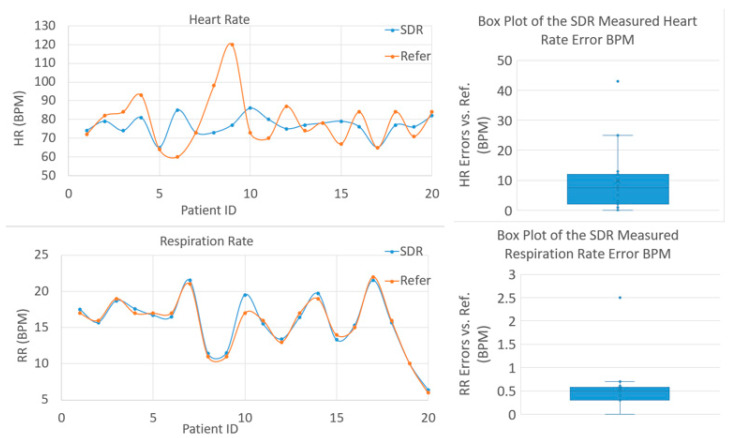
Data analysis on the HR/RR measured by our NCVS sensor system vs. the references on 20 test cases of real patients during the clinical trial.

Before a test is started, a nurse will collect basic information such as age/gender/weight/height, etc. of the patients, then a nurse will measure the patient’s HR with their medical equipment to obtain the reference HR. Next, we run our test on the patients to obtain their HR/RR, during a test. A researcher and/or a nurse needs to manually count the RR by observing the patient’s chest movement to obtain the reference RR. Since it is a clinical trial on real patients, it is very hard to obtain approval from all the patients to wear a respiration belt or an ECG sensor with leads; therefore, manual counting is used to obtain the reference RR data. We have tested in both our engineering lab and also in the TTUHSC clinics on student volunteers, totaling more than 40 cases, to prove that the manual counting of RR is accurate and reliable, as the RR data is almost always identical to the RR data obtained from the respiration belt. Figure 9 shows the test results from 20 test cases in the clinical trial. The NCVS system’s measured HR and reference HR are not always as well-matched as in the lab testing phase, but the RR data matches very well, as is indicated by the error statistics of the boxplots in Figure 9 (Median and STDEV of errors for HR are 7.5/10.7 and for RR are 0.5/0.48, respectively). There are several reasons that could explain why the clinical trial HR monitoring does not perform as accurately as it does in a lab setting. Firstly, the reference HR and NCVS monitored HR tests are not taken simultaneously during the clinical trial testing. Secondly, the room size and surroundings are different in the two settings, which may contribute to the HR error. The smaller room size of the clinical trial room may cause the wall reflection of the signal to be stronger, which degrades the HR accuracy of the system. The surroundings of the clinic room are noisier compared to the engineering lab room, as there are more electrical devices such as PCs, routers, and a refrigerator inside, and there are also people and clinicians walking by the room with its door open; all these vibrations might be detected by the system, thus causing the HR accuracy degradation. Additionally, as is shown in Figure 9, the largest single case error comes from one particular patient who had an HR of 120 BMP for the reference heart rate, while the NCVS system measured an HR of 77 BPM HR. This outlier case might suggest that when performing measurements on a patient who has very high HR, the NCVS system might produce inaccurate HR measurement data. One reasonable explanation for this outlier case is that the upper limit of the cutoff frequency setting on the HR filter in the NCVS system may be too low, as it was set at 2 Hz (i.e., 120 BPM); thus, the high HR signal at 2 Hz is accidently filtered out, and the system might pick up some other signal frequency as the HR. In the future, we plan to increase this HR filter higher cutoff limit to avoid these outlier cases when we resume the clinical trials at TTUHSC after the COVID-19 situation has quieted down further. However, more investigations are needed to examine this case further, and we would need to collect more patients’ data for detailed analysis. Note that the measured RR data from the NCVS system match very well against the reference RR for all cases, as the boxplot of the RR error rate shows a tight and small error distribution (Median error < 0.5 BPM). This excellent agreement is close to what we have found in the lab testing results, strongly indicating the reliability and robustness of the system to produce accurate RR data from NCVS monitoring on actual patients who are sitting on a chair in a clinic. We have thus planned to further use our NCVS system to monitor actual patients’ vital signs when they are lying on beds in a ward, in order to expand the system’s potential clinical usefulness for COVID-19-like scenarios.

**Figure 10:**
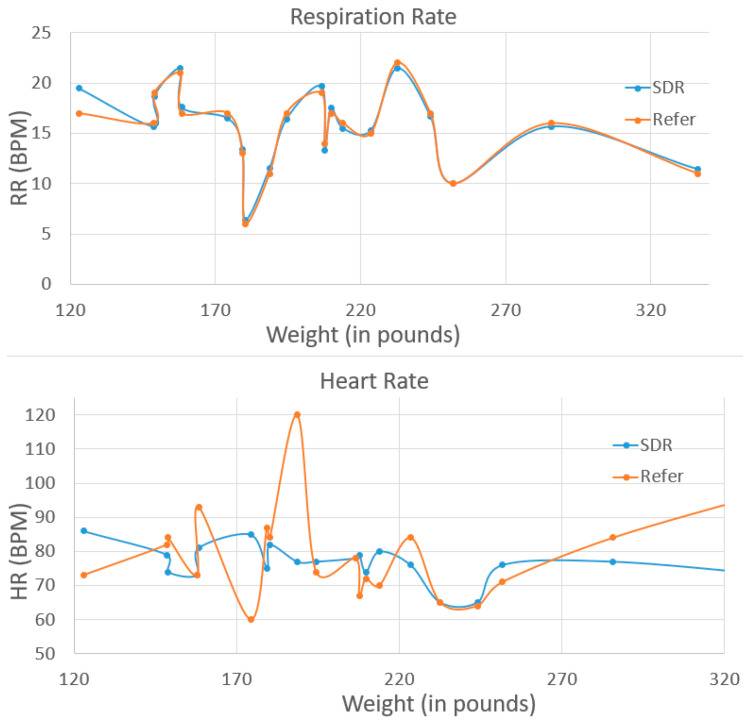
Clinical trial test data on 20 cases sorted by weight.

**Figure 11:**
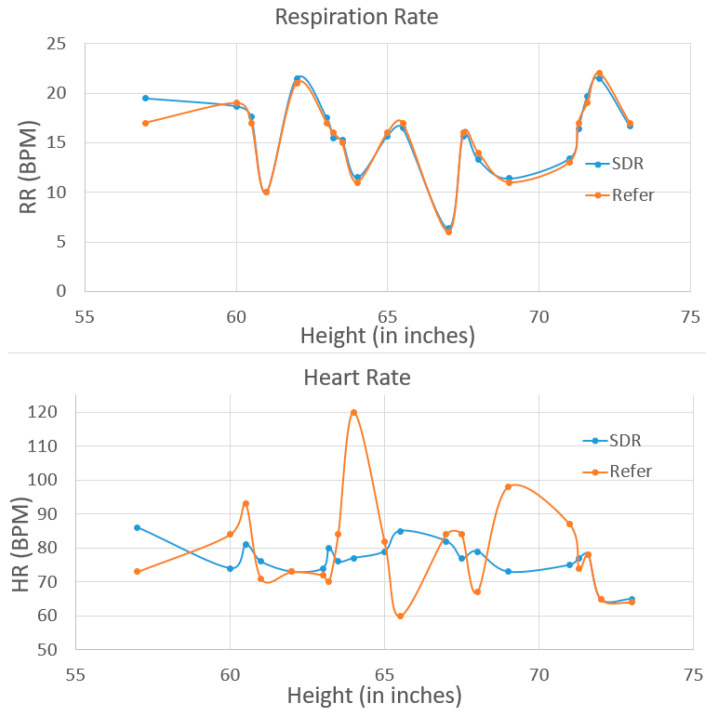
NCVS clinical trial test data on 20 cases sorted by height.

**Figure 12:**
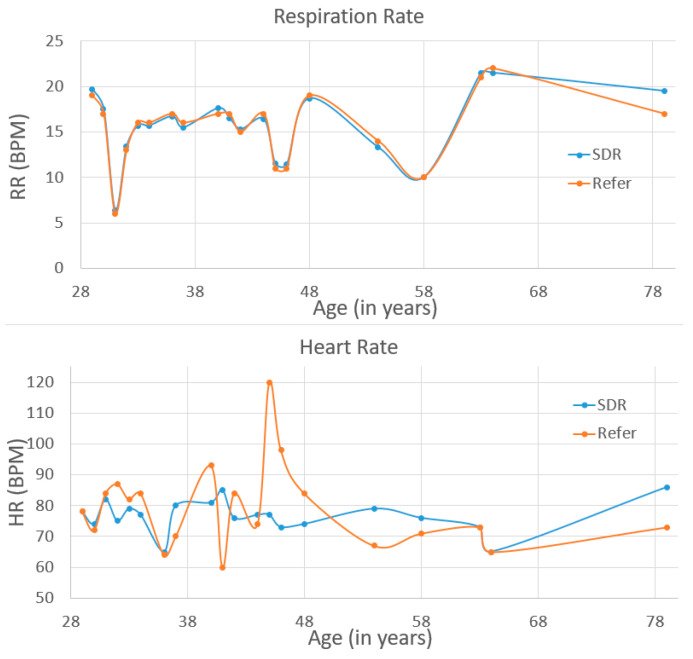
NCVS clinical trial test data from 20 cases sorted by age.

This is the first time our group has used this SDR-enabled NCVS system to test on the real patients, as we want to investigate the feasibility of the system and its robustness for various patients in the real world. For example, it is known that bones and fat will partially absorb/reflect the RF signal, and in this case, how a patient’s body type might influence the system performance is interesting to study. We are measuring and mindful of the patient’s height and weight and age differences to account for chest wall thickness variability and to assess the potential significance of motion artifacts and posture effects. Therefore, the testing results are sorted by weight, height and age correspondingly, as shown in Figure 10, Figure 11 and Figure 12. In the future, a larger group study will be needed to analyze all the relevant data with better statistics. The patients tested had a weight between 123 lbs and 285.8 lbs, height between 4′9” and 6′1” (i.e., 57 inches to 73 inches), and age between 29 and 79. As weight/height/age increased, the mismatch of the NCVS system measured data and reference data did not get any worse or better for either the respiration rate and heart rate measurements. Moreover, the error rate of HR and RR did not show clear signs of increasing or decreasing vs. the weight, height or age. Thus, the data suggest the system’s performance was not noticeably affected by the patient’s body type, height and age within the reported range.

**Figure 13:**
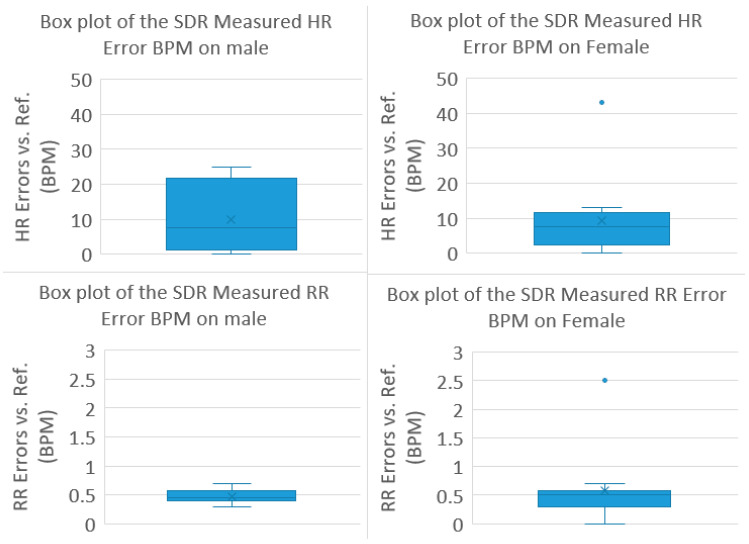
NCVS clinical trial test data from 20 cases as classified by gender.

The clinical trial testing data is also classified according to patients’ gender to study the relationship between the gender and the NCVS monitoring errors. Interesting, according to Figure 13, it appears the NCVS system is more likely to record higher HR errors when testing on the male subjects (Median error 7.5, STDEV 10.3 BPM) vs. on the female subjects (Median error 7.5; STDEV 11.4 BPM), but the statistics on the Median and STDEV appears to be quite similar between the two sexes. As we have 8 male patients and 12 female patients, and the outlier case with the largest HR error as discussed in Figure 8 is also from a female patients’ data, we can purposely remove this outlier data point and then the female patients’ Median HR error is reduced to 7 BPM with a considerably tighter STDEV of 4.6. Therefore, more patients’ data need to be collected to see if there exists a real bias on higher NCVS HR monitoring errors on male patients. Finally, the measured RR errors for both male and female subjects are similar, concentrated around 0.5 BPM with small STDEV (0.12 and 0.63 BPM, respectively), which indicates that the NCVS system’s monitoring accuracy on RR does not show noticeable difference according to a patient’s gender, and it may be highly reliable for real-time monitoring of the RR for various clinical applications, such as monitoring people with suspected COVID-19 who are quarantined at home or in a quarantine hotel. 

## 6. Conclusions

The latest development and clinical testing of this novel SDR-based NCVS monitoring system were presented in this paper. The pre-clinical validation process of lab testing on different student volunteers showed that with proper setup and optimization of the system parameters settings (e.g., on IF and TX/RX gain pair, etc.), the system has the capability to monitor a subject’s real-time HR and RR remotely at 1–2 m away with high accuracy, within 3/0.5 BPM, respectively. The subject’s posture can affect the system’s performance, as we have discovered: having “Bad Posture” during the test might result in generation of noisy I/Q/Arctan channel waveforms, which then decreases the output data accuracy. We then move into the clinical testing phase of this NCVS monitoring system at TTUHSC after receiving an IRB approval. From the analysis of the available clinical trial data on 20 real patients, we found the SDR-based NCVS system has achieved reasonable accuracy for HR and RR monitoring, with Median HR/RR errors of 7.5/0.5 BPM, and STDEV of HR/RR errors of 10.7/0.48 BPM, respectively. One patient’s HR showed as a clear outlier possibly due to our DSP filter cutoff frequency setting, and if we discard that particular HR data point, the Median/STDEV of the HR errors among 19 patients will be significantly reduced to 7/7.5 BPM, respectively. After sorting/classifying the clinical trial data according to patients’ weight, height, age, and gender, we did not find strong evidence to suggest these parameters affected the NCVS-measured HR/RR accuracy of the patients in a significant manner. However, for RR monitoring, all patients have exhibited excellent monitoring accuracy, with errors less than ~0.5 BPM. This SDR-enabled novel NCVS monitoring system has thus successfully demonstrated its clinical capability in remote monitoring of vital signs on real patients, which has never been reported in the literature before, to the authors’ best knowledge. We believe that the adoption of this type of portable SDR-enabled NCVS system can help combat COVID-19 and other highly infectious diseases by providing a means to monitor non-ICU patients without contact and for telemedicine use, and it can be attractive for various other clinical applications, such as for wireless acute-care, continuous NCVS monitoring for at-home patients in quarantine, for rural healthcare and wireless assisted living, etc.

## Figures and Tables

**Table 1:** HR and RR error rates comparison among different NCVS sensing methods.

Ref.	Methods	Frequency Range	Signal Processing Algorithms (in Order)	HR Error Rate (BPM)	RR Error Rate (BPM)
[1]	Visible Light Sensing	300−750 THz	Hann Smoothing + Fast Fourier Transform (FFT)	<5	<1
[2]	FMCW Radar	77−81 GHz	FFT + Phase Extraction + Bandpass Filtering	<8.5	<1.8
[3]	UWB Radar	3.1−4.8 GHz	Moving Target Indicator (Filter) + Max. Distance Gate Selection + Variational Mode Decomposition	<5	1.8
[9]	SDR-based CW Radar System	5.8 GHz	Quadrature Radar Architecture + FFT	N/A	<0.6
[10]	SDR-based CW Radar System	2.4 GHz	Time-of-Arrival Method + FFT	N/A	<0.6
[11]	SDR-based CW Radar System	1/1.5/2 GHz	FFT	<4	<1
This work	SDR-based CW Radar System	2.4 GHz	Arctangent Demodulation + Frequency downconverter + Autocorrelation + FFT	<3	<0.5

## Data Availability

Data is unavailable due to privacy or ethical restrictions.
